# Epidemiological Study of Pathogens in Spontaneous Bacterial Peritonitis in 2017–2024—A Preliminary Report of the University Hospital in South-Eastern Poland

**DOI:** 10.3390/microorganisms12051008

**Published:** 2024-05-17

**Authors:** Jolanta Gruszecka, Rafał Filip

**Affiliations:** 1 Institute of Health Sciences, Medical College of Rzeszow University, 35-310 Rzeszow, Poland; jagrusz@onet.pl; 2 Department of Clinical Microbiology, Clinical Hospital No. 2, 35-301 Rzeszow, Poland; 3 Faculty of Medicine, Medical College of Rzeszow University, 35-959 Rzeszow, Poland; 4 IBD Unit, Department of Gastroenterology, Clinical Hospital No. 2, 35-301 Rzeszow, Poland

**Keywords:** liver cirrhosis, spontaneous bacterial peritonitis, ascites, nosocomial infections, non-hospital infections

## Abstract

Spontaneous Bacterial Peritonitis (SBP) is a serious complication and a common cause of death in patients with liver cirrhosis. Between January 2017 and March 2024, a retrospective study was conducted involving 302 patients (>18 years old) with ascites treated at a tertiary referral center in south-eastern Poland. Microbiological analysis of the ascitic fluids was performed in all patients. The presence of microorganisms was found in samples from 17 patients, and 21 pathogens were isolated, including 15 Gram-positive bacteria and 6 Gram-negative bacteria. *Staphylococcus epidermidis*, MRCNS (methicillin-resistant coagulase-negative staphylococci, resistant to all beta-lactam antibiotics: penicillins, penicillins with beta-lactamase inhibitor, cephalosporins and carbapenems) was the main pathogen detected (19.05%, 4/21), followed by *Enterococcus faecalis* (9.52%, 2/21), *Enterococcus faecium* (9.52%, 2/21), *Staphylococcus haemolyticus*, MRCNS (4.76%, 1/21), *Streptococcus mitis* (9.52%, 2/21), *Streptococcus parasanguinis* (9.52%, 2/21), *Micrococcus luteus* (4.76%, 1/21) and *Bacillus* spp. (4.76%, 1/21). The following Gram-negative bacteria were also found in the specimens examined: *Escherichia coli*, ESBL (extended-spectrum β-lactamase producing *E. coli*) (4.76%, 1/21), *Escherichia coli* (4.76%, 1/21), *Pseudomonas aeruginosa* (4.76%, 1/21), *Klebsiella oxytoca* (9.52%, 2/21) and *Sphingomonas paucimobilis* (4.76%, 1/21). Gram-positive bacteria caused nosocomial infections in nine patients with SBP, Gram-negative bacteria caused nosocomial infections in two patients. In six patients with SBP, community-acquired infections caused by Gram-negative bacteria were found in three cases, Gram-positive bacteria in two cases, and in one case, community-acquired infection was caused by mixed Gram-positive and Gram-negative. Bacteria isolated from patients with hospital-acquired SBP showed higher drug resistance than those found in patients with non-hospital SBP. Bacterial infections in cirrhotic patients with complications may be responsible for their deteriorating health. Prompt intervention is critical to reducing mortality.

## 1. Introduction

Bacterial infections are one of the most important causes of morbidity and mortality in patients with liver cirrhosis. It is also estimated that between 25% and 46% of acute hospital admissions due to decompensation in chronic liver disease (CLD) may be attributed to bacterial infections, out of which, spontaneous bacterial peritonitis (SBP) seems to be the most common (up to 27%) and most severe one [1,2]. Other commonly observed bacterial infections in cirrhotic patients include urinary tract infections (20–22%), respiratory tract infections (15–19%) and bacteremia (12%) [3]. There is a variety of factors associated with SBP development, yet most clinicians agree that the primary underlying pathogenesis of SBP is the bacterial spread from the gut to the lymph nodes in an immunocompromised patient [4]. Generally, Gram-negative bacteria are considered the main causative agents of SBP, with *Escherichia coli* and *Klebsiella* spp. being the most frequently isolated from ascitic fluid [5]. Still, the profile of bacteria may vary according to the geographical region. Moreover, they may be different even between individual hospitals within the selected country. For example, ESBL-producing Enterobacterales dominate in Eastern Europe and Asia, while methicillin-resistant *Staphylococcus aureus* (MRSA) and vancomycin-resistant *Enterococcus* (VRE) are most commonly isolated in the US and South America, carbapenemase-producing *Klebsiella pneumoniae* isolation is increasing in Italy [6,7]. The important aspect herein is that currently hospital-acquired, multi-drug resistant bacteria are more often found in cultures developed from samples in all geographical regions, which in turn contributes to less effective treatment and worse prognosis [6]. Although SBP is rarely observed in patients without liver cirrhosis, neoplastic, autoimmune and portal vein thrombosis as well as cardiac and renal insufficiency—related ascites infections—have also been described [8,9,10,11].

The mortality of SBP in hospitalized patients ranges from 10% to 46%, therefore prompt diagnosis and appropriate treatment are of great importance when managing patients who acquire SBP in various clinical settings [4,8].

Consequently, it is essential to recognize the antimicrobial resistance pattern of isolated bacteria in order to introduce the targeted antibiotic therapy as quickly as possible.

The aim of this study was to perform a retrospective analysis of the frequency of antibiotic resistance patterns of bacterial species isolated from ascitic fluid of patients with liver cirrhosis and spontaneous bacterial peritonitis in a tertiary referral hospital in Rzeszow, southeastern Poland. European guidelines for empirical therapy apply in Poland.

## 2. Methods 

The study was approved by the Bioethics Committee of the Regional Medical Chamber (Resolution No. 88/B/2020 of 24 September 2020). Pursuant to Polish law, patient consent was not required, due to the retrospective nature of the study.

The test results of adult patients admitted and subsequently treated between 2017 and 2024 in a tertiary referral center in south-eastern Poland were reviewed. The hospital has 840 beds, and approximately 80,000 patients are treated there every year. Patients in the hospital were identified by their national identity card and PESEL (Polish Resident Identification) number. The data of all hospitalized patients used for analysis were retrieved from the hospital’s electronic medical records. The IT system operating (AMMS, Asseco Poland S.A., version 6.05.03) in the hospital is under the constant supervision of IT specialists working in the IT section, which is part of the hospital structure. Hospital IT specialists systematically introduce new, up-to-date versions of the software and ensure the protection of personal data. Therefore, a register of all patients staying in the hospital is created on an ongoing basis. All diagnostic laboratories and facilities are also included in the structure of this IT system, which means that all tests are available in the system at the moment they are ordered and then accepted for implementation until the final result is obtained, but only for authorized persons.

The indications for ascitic fluid culture in patients with a known cause of cirrhosis were the following: suspected spontaneous peritonitis or routinely performed culture during decompressing paracentesis. In patients with an unknown cause of ascitic fluid in the peritoneal cavity, bacteriological examination was a routine component of the diagnostic and treatment process.

Peritoneal fluid was collected into bottles with media (at room temperature) allowing aerobic and anaerobic culture, as well as microbial growth detection. Immediately after biological sample collection, the bottles were transferred to the microbiology laboratory located on the hospital grounds. They were then placed in a BactAlert VIRTUO (bioMérieux, Marcy-l’Étoile, France) device for culture and monitoring of bottles with blood and other physiologically sterile body fluids for incubation and periodic readings [12,13,14]. The bottles were incubated in the device for 5 days or less if microbial growth was obtained beforehand [15]. Positive samples were seeded on Columbia Agar with 5% Sheep Blood and MacConkey Agar with Crystal Violet (from aerobic cultures) or Columbia Agar with 5% Sheep Blood, MacConkey Agar with Crystal Violet and Schaedler Agar with 5% Sheep Blood (from anaerobic cultures) (ThermoFisher Scientific, Winsford, UK). Columbia agar plates with 5% sheep’s blood and MacConkey agar plates with crystal violet were incubated for 24 h at 37 °C, and Schaedler agar plates with 5% sheep’s blood were incubated for 48 h at 37 °C.

Identification of microorganisms cultured from biological samples collected from eligible patients was performed by MALDI-TOF (matrix-assisted laser desorption/ionization) mass spectroscopy (MS) using a VITEK MS automated mass spectrometer (bioMérieux, Marcy-l’Étoile, France) [16,17]. MS allows for reliable identification of human pathogens, as well as zoonotic and environmental microorganisms [18]. It involves measuring the mass-to-charge (m/z) ratio of ions produced during ionization of the tested molecules. Matrix-assisted laser desorption/ionization (MALDI) is used as the ionization method. Only a small amount of biological material (usually 1 colony) is needed for identification, and the entire biotyping can be carried out in a short time of several minutes after colony collection, which is a significant advantage over standard biochemical methods. Biotyping also works well for mixed infections [19]. Rapidly obtaining reliable results allows for immediate implementation of appropriate patient management [17,18,20].

The drug resistance profile of the cultured and identified microorganisms was determined using the disc diffusion method or by means of a VITEK2 (bioMérieux, Marcyl’Étoile, France) automatic system for the identification and determination of susceptibility, according to EUCAST (European Committee on Antimicrobial Susceptibility Testing) [21].

Criteria for inclusion: age > 18 years, hospital admission; clinical features of ascites; and absence of a secondary cause of infection (including perforated intra-abdominal abscess determined by clinical criteria or radiographic or surgical findings or evidenced by autopsy).

Criteria for exclusion: age < 18 years; ascites secondary to heart failure/circulatory insufficiency; extrahepatic malignancies; no available data on ascitic fluid; non-cirrhotic portal hypertension and source of infection additional to SBP.

Non-hospital infections were defined as an infection diagnosed within the first 48 h of hospital admission, while a diagnosis made more than 48 h after the start of hospitalization was defined as a nosocomial infection [22,23].

Statistical analysis was performed using PASW Statistics, version 18.0, from IBM (Armonk, New York, NY, USA).

## 3. Results

A total of 302 patients with ascites were involved in the study. The majority of study participants (69%) were men. The age range of the patients studied was 30 to 93 years for women and 27 to 89 years for men. The average age of the patients was 56.95 ± 15.6 years, and 182 patients (60%) were under 65 years old.

The characteristics of the patients are presented in Table 1.

**Figure 1:**
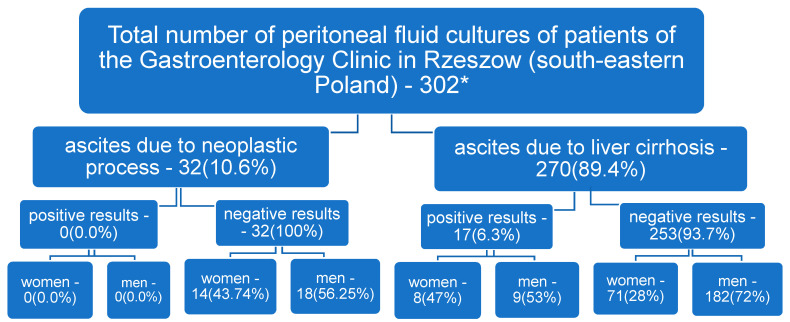
Flowchart of study population of patients of the Department of Gastroenterology in Rzeszow (south-eastern Poland) * patients with other causes of ascites (e.g., cardiac-related) were excluded from the analysis.

In the study population, 270 cases (89.4%, 270/302) were diagnosed with ascites due to cirrhosis and in 32 cases (10.6%, 32/302) ascites due to neoplastic process. Alcohol (92.2%, 249/270) was the leading cause of liver cirrhosis, followed by hepatitis C- and B (HCV, HBV)-related cirrhosis (5.2%, 14/270), and nonalcoholic-steatohepatitis (NASH)- related cirrhosis (2.6%, 14/270). Bacterial growth in this group of individuals was demonstrated in 17 (6.3%, 17/270) samples of ascitic fluid, subjected to microbiological diagnosis, 47% (8/17) of the culture positive ascitic fluids were observed among female participants [Figure 1].

Four fluid samples were diagnosed with mixed growth, bringing the total number of isolated bacteria to twenty-one, 71.4% (15/21) of the pathogens were Gram-positive bacteria. The samples of ascitic fluid examined showed the presence of *Staphylococcus epidermidis* MRCNS 19.04% (4/21), followed by *Enterococcus faecalis* 9.5% (2/21), *Enterococcus faecium* 9.5% (2/21), *Staphylococcus haemolyticus* MRCNS 4.76% (1/21), *Streptococcus mitis* 9.5% (2/21), *Streptococcus parasanguinis* 9.5% (2/21), *Micrococcus luteus* 4.76% (1/21) and *Bacillus* spp. 4.76% (1/21). Gram-negative pathogens were detected in six samples: *Pseudomonas aeruginosa* 4.76% (1/21), *Escherichia coli* ESBL 4.76% (1/21), *Escherichia coli* 4.76% (1/21), *Klebsiella oxytoca* 9.5% (2/21) and *Sphingomonas paucimobilis* 4.76% (1/21). Of the 21 pathogenic bacteria cultured, about 24% (5/21) were methicillin-resistant coagulase-negative staphylococci [Table 2]. Gram-positive bacteria caused nosocomial infections in nine patients with SBP, while Gram-negative bacteria caused nosocomial infections in two patients [Table 2 and Table 3]. One patient with SBP and non-hospital infection had *Streptococcus mitis* 4.76% (1/21) and *Pseudomonas aeruginosa* 4.76% (1/21) isolated, while two patients with non-hospital infection and SBP demonstrated *Staphylococcus epidermidis* MRCNS 9.5% (2/21). Subsequent samples of ascitic fluid from patients with SBP and community-acquired infection, subjected to microbiological analysis, revealed the presence of *Klebsiella oxytoca* 9.5% (2/21) and *Sphingomonas paucimobilis* 4.76% (1/21) [Table 2 and Table 3]. Information on the seasonality of occurrence of nosocomial and community-acquired infections is presented in Table 1.

The remaining 32 cases, 10.6%, 32/302, were found to have ascites due to a neoplastic process. No bacterial growth was observed in cultures of peritoneal fluid collected from this patient group [Figure 1].

Bacterial species isolated from nosocomial and community-acquired spontaneous bacterial peritonitis is shown in Table 3. Patients were treated in accordance with the results of microbiological tests—Table 2.

## 4. Discussion

Body fluids are important in transporting nutrients as well as waste products, regulating body temperature and assessing the respiration process. Naturally, body fluids are sterile under normal circumstances, and the presence of microorganisms indicates an infection [24].

The peritoneal cavity is a sterile site in which no bacteria or any microbes are present as commensals in a healthy state [24]. Any microbe isolated from this site is considered a significant pathogen. The bacteria responsible for peritoneal fluid infection may vary due to various sociodemographic, clinical or medical and behavioral factors related to personnel [22,25,26,27].

In our present study, the overall bacterial infection rate of the peritoneal cavity was 6.3% among patients with ascites with underlying liver cirrhosis.

In most cases, translocation of intestinal bacteria and their product is the major clinical source of peritoneal infections by reduction of intestinal motility, alteration of the gut’s barrier function, and local immune responses [24]. The following Gram-positive bacteria *Enterococcus* spp. were isolated in this study: *E. faecalis* in two samples and *E. faecium* also in two samples of peritoneal fluid examined. This represents 19.05% (4/21) of all bacteria found. According to the literature, *Enterococcus* spp. are found in the intestines of humans and animals. *E. faecalis* is by far the most common (80–90%) isolated species [28]. Other intestinal bacteria, *E. coli* (4.76%, 1/21) and *E. coli* ESBL (4.76%, 1/21), were also present in the ascitic fluid samples tested.

In a study conducted in Egypt, from April 2018 to February 2020, the majority of isolated pathogens were Gram-positive cocci and Gram-negative bacilli—64.3% and 35.7% of all microorganisms found, respectively [1]. Our presented results are similar, Gram-positive bacteria dominated among pathogens, accounting for approximately three quarters of all isolates. The following Gram-negative microorganisms were found in six samples: *Klebsiella oxytoca* 9.52% (2/21), *Sphingomonas paucimobilis* 4.76% (1/21), *P. aeruginosa* 4.76% (1/21), *E. coli* 4.76% (1/21) and *E. coli* ESBL 4.76% (1/21).

The ESBL mechanism occurs in bacteria synthesizing β-lactamases with an extended spectrum of substrates, which makes them resistant to β-lactam antibiotics, most often in Gram-negative bacteria [29,30].

Historically, the main causative agents of SBP have been Gram-negative bacteria, with E. coli and Klebsiella spp. being the most common [5]. Over the past few decades, there has been a shift in the epidemiology of infections in patients with liver cirrhosis, with an increasing prevalence of Gram-positive and multidrug-resistant bacteria diagnosed [26,31].

*S. epidermidis*, also found in our study, is the most commonly isolated bacterial species from human body surfaces. The increase in the incidence of infections involving *S. epidermidis*, observed over the past several years, may be due to the increasingly widespread use of catheters, implants, vascular ports or prostheses (e.g., joint prostheses) in medical treatment, which may constitute vectors for transmission of this bacterium. Problems associated with the course of infections involving *S. epidermidis* include the growing phenomenon of antibiotic resistance. The pathogenicity of *S. epidermidis* is mainly related to the production of agents that enable adhesion to inanimate surfaces and tissues, and also protect against the host immune system [32].

Based on the analysis of our results, we found that out of 17 patients with positive peritoneal fluid cultures, eleven had hospital-acquired infections, while six cases were non-hospital infections. In our study, Gram-positive bacteria predominately caused nosocomial infections, and Gram-negative bacteria were community-acquired.

The latest guidelines from the European Association for the Study of the Liver (EASL) recommend that empiric treatment of SBP in patients with liver cirrhosis and ascites must distinguish between in-hospital and non-hospital SBPs [33].

Gram-positive cocci were the most common bacteria in culture-positive SBP cases in a study conducted at an Athens hospital between 2008 and May 2011 [34]. Another study of a large group of patients in France also found a clear predominance of Gram-positive bacteria, and they accounted for 70% of pathogens cultured from hospital-acquired infections, including MRSA (24.8%). Microorganisms isolated from patients with nosocomial infections showed greater drug resistance than those from patients with community-acquired infections [23].

SBP has a staggering mortality rate of 40–70% in cirrhotic patients, and this further rises to 80% for patients who develop septic shock [35,36,37]. The overall prevalence of SBP in cirrhosis is 17.12%, with high variability in SBP prevalence in different geographic regions. A four-times higher SBP rate was observed in Africa compared to North America (44.54% vs. 10.81%). The aggregate prevalence of SBP in the Asia–Pacific region was about 14% and about 18% in Europe. The mortality rate was found to be 30.61% [35]. In Poland, the overall prevalence of SBP in liver cirrhosis is less than 15% and 30-day mortality is estimated at 10–50% [6,35].

A prospective evaluation of two series of hospitalized patients with uncompensated cirrhosis was conducted. The first series, carried out in 2011, included 1146 patients from northern, southern and western Europe. In 2017–2018, a second series of 883 patients from eastern, southern and western Europe was studied to detect potential epidemic changes. SBP (n = 130) and UTI (n = 111) were the most commonly confirmed infections in the entire series and in southern and western European patients. The distribution of SBP incidence was as follows: 130 (25.0%) in total, including 14 (18.1%) in northern Europe, 54 (25.1%) in southern Europe and 62 (26.1%) in western Europe. Bacterial isolation was similar in in-hospital and non-hospital infections (53% versus 49%). In total, 28.1% of the organisms detected in the study showed multi-drug resistance, the most common being *E. coli* ESBL, followed by *S. aureus* MRSA [38,39,40].

Multidrug-resistant bacterial infections are a global and growing healthcare problem in decompensated cirrhosis across Europe [36].

In our presented study, 28.6% (6/21) of detected microorganisms showed multi-drug resistance (5/21, 23.84%—Gram-positive cocci; 1/21, 4.76%—Gram-negative bacilli). Of the 21 microorganisms isolated from the ascitic fluid, 14 (66.67%, 14/21) were the cause of a hospital-acquired infection.

Our study has several limitations. This was a single-center, retrospective study with a relatively small sample size, which may have reduced its statistical power. However, the strength of the study is the fact that it is the only epidemiological study in this part of Europe of culture results and antibiotic resistance of microorganisms isolated from ascitic fluid in patients with hepatic cirrhosis.

## 5. Conclusions

In our study with liver cirrhosis patients, the rate of positive ascitic fluid cultures in SBP was 6.3%. Among the pathogens detected, 71.4% were Gram-positive, and 23.8% were methicillin-resistant coagulase-negative *Staphylococci*. Gram-negative bacteria accounted for 29.6%, and *E. coli* ESBL was found in one sample. Although the presented results give some insight into the local epidemiological situation, multi-center studies will certainly be more representative, and the results could be extrapolated to the whole Polish population.

## Figures and Tables

**Table 1:** Clinical characteristics of study population.

Characteristics of the Patients	Positive Peritoneal Fluid Culture (n = 17)		Negative Peritoneal Fluid Culture (n = 285)	
	Women (n = 8)	Men (n = 9)	Women (n = 85)	Men (n = 200)
Age of patients—arithmetic mean, standard deviation, (years old)	62.5 ± 16 (48–89)	45.4 ± 15 (32–74)	60.7 ± 19.3 (30–93)	59.6 ± 11.6 (29–89)
Hospitalization duration—arithmetic mean, standard deviation, (days)	16 ± 7.9 (8–28)			
Origin of the infection—arithmetic mean, standard deviation, (years old)	community-acquired		hospital-acquired	
	n = 6		n = 11	
	Women (n = 2) 41.5 ± 0.5 (41–42)	Men (n = 4) 54.5 ± 18 (32–74)	Women (n = 6) 60.3 ± 15 (43–82)	Men (n = 5) 51.2 ± 14 (36–73)
	seasonality of occurrence			
	community-acquired		hospital-acquired	
	Summer months (n = 2) 58 ± 16 (42–74)	Winter months (n = 4) 51.7 ± 12.5 (32–63)	Summer months (n = 4) 72 ± 12.7 (51–82)	Winter months (n = 7) 49 ± 11.3 (35–69)
Treatment effect on SBP—arithmetic mean, standard deviation, (years old)	Improvement—14		Death—3	
	Women (n = 8) 62.5 ± 16 (48–89)	Men (n = 6) 61.7 ± 9.1 (50–74)	Women—0	Men-3 34.3 ± 1.7 (32–36)
Drugs administered to patients included in the study	Antibiotics—according to the result of the microbiological test.			
	Proton Pump Inhibitor (PPI)—preparations containing panto-prazole (Pantoprazol, Anesteloc, Controloc, Ozzion) esomepra-zole (Esomeprazol, Esium), omeprazole (Helicid).			
	Steroids—preparations containing glicocorticosteroids: prednizon (Encorton), metyloprednizolon (Metypred, Corhydron), Hydrokortyzon			

**Table 2:** Results of peritoneal fluid culture from patients of the Gastroenterology Clinic in Rzeszow (south-eastern Poland) with diagnosed microorganisms and their drug susceptibility, 2017–2024.

	The Microorganisms Identified n	*S. epidermidis * MRCNS	*S. haemolyticus* MRCNS	*E. faecalis*	*E. faecium*	*K. oxytoca*	*S. mitis*	*S. parasanguinis*	*M. luteus*	*P. aeruginosa*	*S. Paucimobilis*	*B.* spp.	*E. coli * ESBL	*E. coli*
The Number of Microorganisms Sensitive, n		4	1	2	2	2	2	2	1	1	1	1	1	1
AK		1				2			1	1			1	1
ATM										1				
AMC						2							1	1
CAZ						2				1	1			1
CTX						2	1	2						1
CRO							2	2						
CT										1			1	1
CIP						2					1	1		1
E												1		
CXM														1
FEP						2				1				1
GM		1			1	2			1				1	1
IPM				2		2				1	1	1	1	1
MEM						2				1	1	1	1	1
LEV											1	1		
TZP						2				1	1		1	1
TGC		4	1	2	2	2			1				1	1
TM						2							1	1
SXT		3				2			1				1	1
AM				2			2	2						
LZD		4	1	2	2				1			1		
TEC			1	2	2		2	2						
VA		4	1	2	2		2	2				1		
CM		1					2	2						
TE		1							1					
PIP										1	1			
P							1							
PG							1	1						

AK—Amikacin; AM—Ampicillin; AMC—Amoxicillin/Clavulanic Acid; ATM—Aztreonam; FEP—Cefepime; CTX—Cefotaxime; CAZ—Ceftazidime; CM—Clindamycin; CIP—Ciprofloxacin; CRO—Ceftriaxone; CT—Colistin; CXM—Cefuroxime; E—Erythromycine; GM—Gentamicin; IPM—Imipenem; LZD—Linezolid; LEV—Levofloxacin; MEM—Meropenem; P—Penicillin; PG—Benzylpenicillin; PIP—Piperacillin; TEC—Teicoplanin; TE—Tetracycline; TM—Tobramycyna; TZP—Piperacillin/Tazobactam; TGC—Tigecycline; SXT—Trimethoprim/Sulfamethoxazole; VA—Vancomycin. MRCNS—methicillin-resistant coagulase-negative staphylococci (resistant to all beta-lactam antibiotics: penicillins, penicillins with beta-lactamase inhibitor, cephalosporins and carbapenems). ESBL—with Extended Spectrum Beta-Lactamase.

**Table 3:** Bacterial species isolated from nosocomial and community-acquired spontaneous bacterial peritonitis.

Pathogen	Total (n, %)	Hospital SBP (n, %)	Non-Hospital SBP (n, %)	*p*-Value
**Gram-positive**	**15 (71.43)**	**12 (57.14)**	**3 (14.29)**	<0.001
*Staphylococcus epidermidis* MRCNS	4 (19.05)	2 (9.52)	2 (9.52)	=0.001
*Enterococcus faecalis*	2 (9.52)	2 (9.52)	-	=0.064
*Enterococcus faecium*	2 (9.52)	2 (9.52)	-	=0.064
*Staphylococcus haemolyticus* MRCNS	1 (4.76)	1 (4.76)	-	=0.333
*Streptococcus mitis*	2 (9.52)	1 (4.76)	1 (4.76)	=0.064
*Streptococcus parasanguinis*	2 (9.52)	2 (9.52)	-	=0.064
*Micrococcus luteus*	1 (4.76)	1 (4.76)	-	=0.333
*Bacillus* spp.	1 (4.76)	1 (4.76)		=0.333
**Gram-negative**	**6 (28.57)**	**2 (9.52)**	**4 (19.05)**	<0.001
*Pseudomonas aeruginosa*	1 (4.76)		1 (4.76)	=0.333
*Escherichia coli* ESBL	1 (4.76)	1 (4.76)	-	=0.333
*Escherichia coli*	1 (4.76)	1 (4.76)	-	=0.333
*Klebsiella oxytoca*	2 (9.52)	-	2 (9.52)	=0.064
*Sphingomonas paucimobilis*	1 (4.76)	-	1 (4.76)	=0.333

Legend: *p*-values were calculated using Student’s *t*-test.

## Data Availability

The original contributions presented in the study are included in the article; further inquiries can be directed to the corresponding author.
